# Targeting the NLRP3 inflammasome for calcium oxalate stones: pathophysiology and emerging pharmacological interventions

**DOI:** 10.3389/fphys.2025.1614438

**Published:** 2025-06-03

**Authors:** Andrew M. Boldt, Francesca Di Sole

**Affiliations:** Physiology and Pharmacology Department, Des Moines University, Des Moines, IA, United States

**Keywords:** calcium oxalate stones, kidney stone disease, NLRP3 inflammasome, chronic kidney disease, renal inflammation

## Abstract

Kidney stone disease (nephrolithiasis) is a widespread condition affecting millions worldwide, with its prevalence rising due to dietary changes, obesity, and climate-related factors. The formation of kidney stones is driven by urinary solute supersaturation, metabolic abnormalities, and environmental influences. Calcium oxalate stones, the most common type, result from hypercalciuria, hyperoxaluria, and hypocitraturia, often exacerbated by high dietary protein intake and hormonal imbalances such as hyperparathyroidism. A significant complication of kidney stones is their association with chronic kidney disease (CKD). Recurrent stone formation contributes to renal scarring, urinary obstruction, and inflammation, ultimately leading to long-term kidney damage. This review explores the pivotal role of the NLRP3 inflammasome in kidney stone-related inflammation. Activated by calcium oxalate crystals and oxidative stress, NLRP3 triggers the release of pro-inflammatory cytokines (IL-1β and IL-18), exacerbating renal injury and fibrosis. Persistent NLRP3 activation is linked to CKD progression and an increased risk of end-stage renal disease. Emerging therapies targeting NLRP3 offer potential strategies to mitigate kidney stone-induced inflammation and CKD progression. Direct inhibitors such as MCC950 and CP-456773 block inflammasome activation, reducing inflammatory cytokine release. Indirect approaches, including atorvastatin and phenylbutyric acid, address oxidative stress and mitochondrial dysfunction to lower stone formation risk. While these treatments show promise in preclinical studies, further research is needed to validate their clinical efficacy. Future studies should focus on optimizing NLRP3-targeted therapies, assessing their long-term effects on kidney stone prevention and CKD progression. Combining NLRP3 inhibitors with antioxidants may enhance renal protection, providing new avenues for therapeutic intervention.

## 1 Introduction: overview of kidney stones and their clinical significance

Kidney stone disease, also referred to as nephrolithiasis or urolithiasis, occurs when urinary solutes crystallize, forming aggregates within the urinary tract (Shastri et al., 2023). This global health issue affects approximately 1 in 11 individuals in the United States and is influenced by genetic predisposition, metabolic disorders, inflammatory bowel disease, hypertension, dehydration, and dietary habits (Hoffman et al., 2021). Treating kidney stones costs more than $2 billion annually in the United States (Pearle et al., 2005).

The prevalence of kidney stone disease has increased significantly, rising from 3.2% in the late 1970s to 8.8% by 2014 (Stamatelou and Goldfarb, 2023). By age 70, kidney stones affect 19.1% of men and 9.4% of women. The male-to-female ratio has narrowed from 3:1 to 2:1, likely due to rising obesity rates, which, along with diabetes, are strongly associated with kidney stones in women (Shastri et al., 2023). These conditions may have reduced the historical gap between sexes, contributing to the more balanced ratio observed in recent years.

Racial and ethnic disparities are evident, with White males having the highest prevalence, followed by Hispanic and Asian populations, and Black individuals the lowest. Globally, men in the United Arab Emirates and Saudi Arabia face the highest risks. In the United States, prevalence increases geographically from North to South and West to East, driven by obesity, diabetes, and heat-related factors that elevate urinary concentration (Crivelli et al., 2021). The incidence is expected to rise further due to climate change (Stamatelou and Goldfarb, 2023).

Kidney stones have a high recurrence rate, with 50% of individuals experiencing a relapse within 5–10 years and 75% within 20 years (Daudon et al., 2018). Risk factors for recurrence include a history of multiple stones, early age of onset, male sex, a family history of kidney stones, elevated BMI, and specific stone compositions (Daudon et al., 2018; Wang et al., 2022).

Beyond causing acute symptoms such as severe pain and hematuria, kidney stones are markers of systemic diseases, associated with increased risks of metabolic syndromes, hypertension, and cardiovascular complications (Jeong et al., 2011; Muschialli et al., 2024). This underscores their broader clinical importance as both a symptom and predictor of systemic health issues.

## 2 Link between kidney stones and chronic kidney disease

Kidney stones can directly contribute to chronic kidney disease (CKD) by causing recurrent urinary tract obstruction, inflammation, and infection. Over time, these processes may lead to scarring of renal tissue, impaired kidney function, and irreversible damage. Additionally, certain types of stones, such as calcium oxalate stones, are associated with metabolic abnormalities like hypercalciuria or hyperoxaluria, which can further strain kidney function (Zisman et al., 2015; Gambaro et al., 2017). Epidemiological studies have consistently shown a higher incidence of CKD in individuals with a history of kidney stones. These studies emphasize that kidney stones not only increase the risk of CKD development but also its progression to severe stages, including end-stage renal disease (ESRD) (Rule et al., 2009; Kim et al., 2024). Factors such as recurrent stone formation, chronic obstruction, and associated urinary infections amplify this risk (Jungers et al., 2004; Gambaro et al., 2017).

## 3 Role of oxidative stress and inflammatory response in kidney stones

Oxidative stress plays a pivotal role in kidney stone formation. An imbalance between reactive oxygen species (ROS) production and antioxidant defenses leads to cellular damage within renal tubules (Du et al., 2014). ROS can enhance calcium and oxalate crystal deposition by damaging epithelial cells and promoting nucleation and aggregation of crystals. Additionally, oxidative stress can alter the expression of proteins that regulate mineralization, further promoting stone growth. Kidney stones elicit a local inflammatory response within the urinary tract. The irritation caused by stone fragments and crystals leads to the activation of immune cells, releasing pro-inflammatory cytokines like interleukin-6 (IL-6) and tumor necrosis factor-alpha (TNF-α) (Wigner et al., 2021)

Chronic inflammation not only contributes to pain and tissue damage but also creates a favorable environment for further stone formation by enhancing crystal retention and growth (Jia et al., 2022; Deguchi et al., 2024; Sun et al., 2024).

## 4 Calcium oxalate stones and the NLRP3 inflammasome

### 4.1 Pathophysiology of calcium oxalate stones

As outlined in the introduction, urinary stone formation is driven by the principle of supersaturation, where urinary solutes exceed their solubility, promoting crystallization and stone development. Several factors contribute to this process, including obesity, genetics, diabetes, and anatomical abnormalities, as also discussed in the introduction. However, diet- and hormone-induced stones are the most common types, with calcium oxalate and calcium phosphate being the two most prevalent compositions (Shastri et al., 2023). Calcium based stones, oxalate and phosphate, have similar risk factors in stone formation such as alkaline urinary pH, hypercalciuria, hyperoxaluria, hypocitraturia, hyperuricosuria, and anatomical abnormalities such as tortuous ureters or medullary sponge kidney (Shastri et al., 2023). These factors interplay with metabolic and environmental influences to promote stone formation.

#### 4.1.1 Calcium oxalate stone formation: Dietary factors

Diet is one the most common cause of calcium oxalate stones. High dietary calcium intake or secondary causes can lead to hypercalciuria which promotes the precipitation of calcium deposits (Pak et al., 2011). These deposits, along with other risk factors such as an alkaline urinary pH, hyperoxaluria, hypocitraturia, and anatomical abnormalities, contribute to the formation of calcium oxalate stones. Secondary causes can further exacerbate hypercalciuria, including increased gastrointestinal calcium absorption, increased bone turnover, or decreased renal tubular reabsorption (Worcester and Coe, 2008). Dietary protein also plays a significant role in stone formation. Increased dietary protein intake raises acids production in the body, which can stimulate bone resorption and result in higher calcium excretion (Goldfarb, 1988). The acidic excretion of uric acid further impairs calcium reabsorption, contributing to hypercalciuria and stone formation. Additionally, the protein-induced hypercalciuric effect may increase urinary sulfate excretion which form calcium-sulfate complexes in renal tubules. These poorly absorbable complexes can perpetuate nephrolithiasis by further promoting stone formation (Goldfarb, 1988; Minisola et al., 2018).

#### 4.1.2 Calcium oxalate stone formation: hormonal causes

Hypercalciuria is often driven by hypercalcemia, which is tightly regulated by parathyroid hormone, and calcitriol. These regulatory mechanisms work together to maintain stable blood calcium levels. However, hormonal abnormalities can disrupt this balance, causing the renal system to improperly reabsorb calcium and instead secrete it excessively, leading to hypercalciuria (Pak et al., 2011; Minisola et al., 2018). In addition to dietary factors, hormonal imbalances contribute to calcium oxalate stone formation, with the most common causes being hyperparathyroidism. This can manifest as primary hyperparathyroidism caused by overactive parathyroid glands excessively producing parathyroid hormone (PTH), or secondary hyperparathyroidism where an exogenous factor such as CKD or vitamin D deficiency stimulates increased PTH production. PTH plays a central role in calcium homeostasis by promoting hypercalcemia. It does it by increasing the synthesis of calcitriol, which enhances intestinal absorption of calcium; and by regulating the renal system to increase calcium reabsorption. Additionally, PTH accelerates bone turnover, releasing calcium into the bloodstream (Coe et al., 1992). These mechanisms collectively elevate both blood and urine calcium concentrations, significantly increasing the risk for calcium-related kidney stones in a state of hyperparathyroidism.

#### 4.1.3 Mechanisms of calcium oxalate stone formation

**FIGURE 1:**
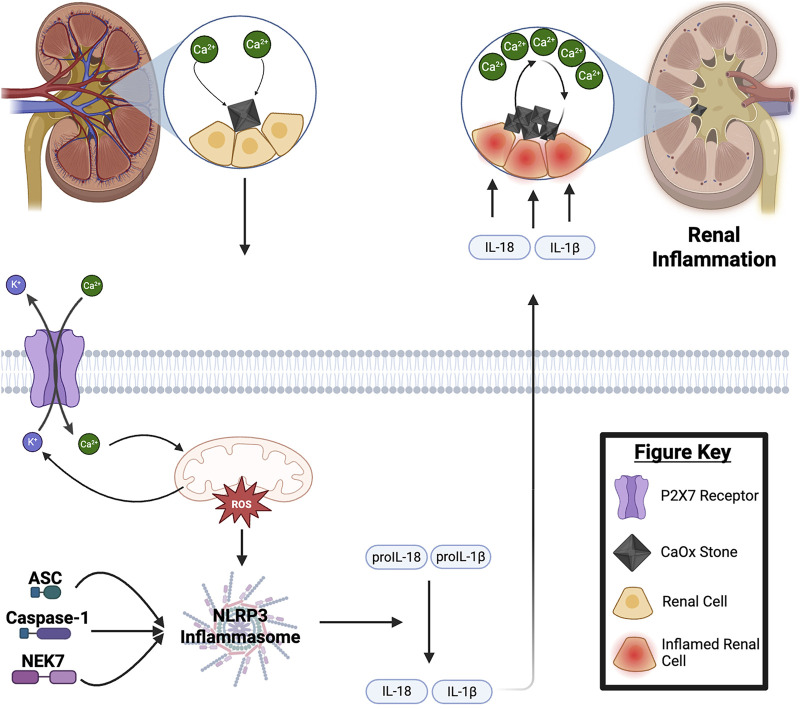
Mechanisms of Calcium Oxalate Stone Formation: NLRP3 Inflammasome and Renal Inflammation. Under conditions of calcium supersaturation, Randall’s plaques form in the renal papilla serving as deposition sites for calcium oxalate (CaOx) stone formation. These crystals accumulate in the tubular lumen of nephrons, while excess extracellular calcium enters renal cells through P2X7 receptors, causing mitochondrial stress and production of radical oxygen species (mtROS). The resulting mtROS triggers the assembly of the NLRP3 inflammasome, which activates cytokines IL-18 and IL-1β. Through paracrine signaling, these cytokines, further stimulate mtROS production and calcium expulsion from neighboring renal cells (Cao et al., 2016; Fong-Ngern et al., 2017; Chaiyarit and Thongboonkerd, 2020). This creates a self-perpetuating cycle of renal inflammation, CaOx crystallization, aggregation, and nucleation which ultimately contributes to kidney damage and stone formation. For a detailed discussion of NLRP3 inflammasome activation mechanisms, please refer to the following review articles (Swanson et al., 2019; Fu and Wu, 2023).

The physical process of calcium oxalate stone formation begins with urinary supersaturation and the retention of crystals in kidney pouches. It is theorized that ulceration at the papillary surface creates a stone nidus, which promotes renal tubular injury and facilitates crystal nucleation (Tsujihata, 2008). This interaction between crystals and cells forms the foundation for stone growth (Tsujihata, 2008). A key side in this process is the formation of Randall’s plaques, subepithelial calcium phosphate deposits that develop under supersaturated conditions (Figure 1). These plaques become embedded in the urothelium, initiating crystal formation (Tsujihata, 2008; Mulay et al., 2013). Once the crystals adhere to renal tubules or papilla, their surface are exposed to further nucleation (Tsujihata, 2008; Leslie et al., 2025). This spontaneous process depends on urinary composition, flow, and pH; then crystal aggregation occurs when calcium oxalate crystals cluster together, driving stone growth (Tsujihata, 2008; Mulay et al., 2013; Leslie et al., 2025). Current research is investigating the role of urothelial cell surface injury and repair. Alterations in cell surface molecular expression might contribute to recurrent stone formation by creating favorable conditions for crystal adhesion and retention (Asselman et al., 2005).

### 4.2 The NLRP3 Inflammasome’s contribution to CKD and associated renal inflammation and damage

The nod-like receptor protein 3 (NLRP3) inflammasome is a polyprotein complex that regulates chronic inflammatory response. Through a multimodal, stepwise process, NLRP3 activation promotes the cleavage of cytokine precursors, such as interleukin (IL)-1β, to trigger an inflammatory response (Schroder and Tschopp, 2010). The activation of NLRP3 leads to the activation of an apoptosis-associated speck-like protein containing a caspase domain (ASC). Once initiated ASC recruits pro-caspase-1 to the inflammasome complex (Fernandes-Alnemri et al., 2007). Caspase 1 is then activated autocatalytically and cleaves pro-IL-1β and pro-IL-18 into their mature, active forms (Fernandes-Alnemri et al., 2007) (Figure 1). Additionally, Caspase-1 cleaves gasdermin D, which forms pores in the plasma membrane, triggering a lytic, pro-inflammatory form of cell death. This process expels more cytokines, further perpetuating the inflammatory response (Fernandes-Alnemri et al., 2007). The activation of IL-1β and IL-18 drives an endothelial response, promoting migration of immune cells to damaged tissue (Fernandes-Alnemri et al., 2007). In the context of renal inflammation, this facilitates tissue damage and dysregulation, particularly in the glomeruli and tubules (Kitching et al., 2005). These disruptions impair renal solute and osmotic regulation. Systemically, IL-1β and IL-18 contribute to hypotension, fever, pain sensitivity, and vasodilation (Lee et al., 2004; Malik and Kanneganti, 2018). Hypotension and vasodilation are particularly significant for renal function, as decreased renal blood flow and prolonged hypoperfusion can lead to reduced glomerular filtration rate (GFR), further perpetuating renal injury (Smarick, 2009; Lankadeva et al., 2022). Thus, the activation of IL-1β and IL-18 by NLRP3 not only causes direct renal cellular damage and inflammation but also exacerbates systemic renal damage and dysfunction.

These mature cytokines are central for inflammation, causing cellular membrane dysfunction, promoting programmed cell death, and inducing ROS, which further exacerbate cellular and DNA damage (Swanson et al., 2019; McKee and Coll, 2020). NLRP3’s activation can occur in response to both infectious or non-infectious processes (Swanson et al., 2019; McKee and Coll, 2020). Once activated, its effects contribute to renal injury, and persistent activation can lead to metabolic disorders and acute kidney injury (AKI) (Zheng et al., 2021). Over time, this persistent inflammation may progress to CKD (Zheng et al., 2021). Prior research has established a connection between NLRP3 expression with CKD. Increasing proteinuria has been found to correlate with NLRP3 accumulation in glomerular podocytes of systemic lupus erythematosus patients with lupus nephritis (Fu et al., 2017; Oliveira et al., 2021). This correlation is also associated with crescentic glomerulonephritis, characterized by vascular necrosis and parietal epithelial cell hyperplasia in the space surrounding the glomerulus, forming crescents and highlights the progressive accumulation of NLRP3 as the disease advances (Islamuddin and Qin, 2024). Additionally, RT-PCR of mRNA levels of NLRP3 and its inflammatory components were higher in the peripheral blood mononuclear cells isolated from hemodialyzed CKD patients compared to healthy subjects (Granata et al., 2015). These findings highlight the correlation of the NLRP3 inflammasome to renal inflammation and injury, as well as its broader role in the progression of renal diseases.

#### 4.2.1 The role of the NLRP3 inflammasome in calcium oxalate stone-induced inflammation and damage

Many factors promote the assembly and activation of NLRP3, as seen in nephrolithiasis and its association with CKD. While nephrolithiasis is known to cause renal inflammation and damage, the specific mechanisms underlying its action remain widely debated. Activation of the NLRP3 inflammasome follows a two-step mechanism: priming and activation. In renal tubular epithelial cells, priming involves upregulation of NLRP3 expression and is triggered by calcium oxalate crystals adhering to or penetrating the plasma membrane (Sun et al., 2015; Sun et al., 2017). This interaction leads to the release of danger-associated molecular patterns (DAMPs), such as ATP (Rumora et al., 2021), which initiate signaling pathways, such as NF-κB, that increase transcription of inflammasome components (Liu et al., 2017).

The second step, activation, involves the assembly and functional triggering of the inflammasome complex. In epithelial cells, this step is typically induced by increased potassium efflux (i.e., a decrease in intracellular K^+^ concentration) through ion channels. Potassium efflux is a well-established trigger of NLRP3 inflammasome activation, whereas the role of calcium flux in this process remains controversial (Huang et al., 2023). Mulay et al. used mouse models to investigate calcium oxalate crystal-induced NLRP3 activation. They proposed that phagocytosis of calcium oxalate crystals, interconnected with potassium efflux, results in NLRP3 activation. To test this, they used Cytochalasin D, an inhibitor of actin polymerization that blocks phagocytosis, which reduced crystal uptake and decreased IL-1β secretion. Furthermore, blocking potassium efflux via high extracellular potassium produced the same outcome, preventing inflammasome activation, an effect similarly observed in monocytes and bone marrow–derived macrophages (Mulay et al., 2013; Munoz-Planillo et al., 2013; Gritsenko et al., 2020). However, cytochalasin D has also been shown to impair potassium efflux directly, suggesting that it may affect both phagocytosis and ion flux (Munoz-Planillo et al., 2013). These findings demonstrate a direct molecular mechanism in which calcium oxalate phagocytosis induces NLRP3 activation, leading to IL-1β production and subsequent renal inflammation. Expanding their investigation, Mulay et al. examined diffuse crystal deposition in calcium oxalate treated mice and its association with renal failure. They observed that mice with calcium oxalate deposition exhibited increased diffuse neutrophil infiltrates and tubular necrosis, characterized by granular casts and transient elevations in plasma creatinine and blood urea nitrogen (BUN) levels (Mulay et al., 2013). This research highlights the acute inflammatory role of NLRP3 activation by calcium oxalate stones, emphasizing its contribution to renal damage and inflammation.

## 5 Pharmacological interventions for kidney stones targeting NLRP3 inflammasome: mechanisms of action and clinical implications

### 5.1 How pharmacological agents inhibit NLRP3 activation and downstream inflammatory pathways

Due to the complex cascade of NLRP3 activation, various inhibition strategies have been developed to target this inflammasome. Whether acting directly or indirectly, upstream or downstream, many pathways can be modulated to prevent NLRP3 activation or inhibit the formation of pro-inflammatory cytokines. Key inhibition targets include caspase-1 activation, blockade of gasdermin D cleavage, inhibition of P2X7 receptor, and use of ROS scavengers (Lamkanfi et al., 2009; Zahid et al., 2019).

The most common inhibition strategies target NLRP3 directly to block its ATPase activity by preventing its ATP binding domain. This inhibits the conformational changes necessary to inflammasome assembly and activation. For example, CY-09, an analog of cystic fibrosis transmembrane conductance regulator (CFTR) channel, lacks CFTR-inhibitory activity and directly interferes with NLRP3 ATP binding, preventing its activation (Huang et al., 2018; Zahid et al., 2019). Therapeutically, CY-09 has shown promising effects in preclinical model of gout, cryopyrin-associated periodic syndromes (CAPS), and type 2 diabetes (Huang et al., 2018; Zahid et al., 2019). Although not yet used in clinical practice, ongoing research aims to evaluate its pharmacological potential in humans.

In addition to direct and indirect NLRP3 inhibitors, various other agents target different components of the inflammasome pathway. A summary of these pharmacological inhibitors, their targets, and proposed mechanisms is provided in Table 1.

**TABLE 1:** Summary of pharmacological agents, their molecular targets, and proposed mechanisms of action in the inflammasome pathway.

Agent	Target(s)	Proposed mechanism
Glyburide	NLRP3 (indirectly)	Inhibits ATP-sensitive K^+^ channels, preventing ASC aggregation downstream of P2X7 (Perregaux et al., 2001; Lamkanfi et al., 2009; Zahid et al., 2019)
CP-424,174	IL-1β	Blocking the formation of mature IL-1β does not increase the extracellular release of the procytokine. (Perregaux et al., 2001)
MCC950 CP-456,773 CRID3	NLRP3 (directly)	Blocks ATPase domain of NLRP3, inhibiting both canonical and non-canonical NLRP3 activation (Coll et al., 2015; Ludwig-Portugall et al., 2016; Zahid et al., 2019; Hochheiser et al., 2022; Xu et al., 2025)
VX-740	Caspase-1 (indirectly)	Modifies caspase-1 catalytic cysteine residue, preserving cleavage of cleaving pro-IL-1 β and pro-IL-18 (Rudolphi et al., 2003; Boxer et al., 2010; Zahid et al., 2019)
1,3-Butanediol	NLRP3	The macrophage phenotype and inflammasome component NLRP3 contributes to nephrocalcinosis-related chronic kidney disease independent from IL-1–mediated tissue injury (Anders et al., 2018)
Necrosulfonamide (NSA)	Gasdermin D (blocks a downstream effect of NLRP3)	Necrosulfonamide acts downstream of NLRP3 by blocking Gasdermin D-mediated pore formation, preventing pyroptosis and the release of IL-1β. It does not affect inflammasome assembly or caspase-1 activation. (Chen et al., 2023)
Atorvastatin	Suppression of ROS, NF-κB, and mitochondrial dysfunction	Inhibiting both the priming and activation phases of the NLRP3 inflammasome (reduction ROS, suppression NF-kB) (Sun et al., 2020)
Polydatin (3,4′,5-trihydroxystilbene-3-β-D-glucoside), an antioxidant extracted from *Polygonum cuspidatum*	ROS production NLRP3	Decreases cytoplasmic and mitochondrial ROS (mtROS), blocking both the priming and activation of the NLRP3 inflammasome. (Liu et al., 2023)
Vitexin	ROS Production NF-κB NLRP3	Suppressing NLRP3 inflammasome activation and downstream pyroptosis. (Ding et al., 2021)

### 5.2 Therapeutic potential of direct and indirect NLRP3 inflammasome inhibitors in kidney stone-related inflammation

#### 5.2.1 Targeting the NLRP3 inflammasome directly

CP-456,773 has emerged as a promising NLRP3 inflammasome inhibitor. In mouse models, CP-456,773 has been shown to suppress IL-1β and IL-18 levels demonstrating efficacy comparable to genetically NLRP3-deficient mice (Joshi et al., 2015; Primiano et al., 2016). Studies comparing wild-type (WT) mice to mice on an adenine-enriched diet (which promotes calcium oxalate stone formation) revealed that CP-456,773 treatment significantly lowered serum levels of BUN, creatinine, indicating reduced renal injury. However, it did not prevent crystal formation as both treated and untreated mice exhibited intrarenal crystal deposits (Joshi et al., 2015; Primiano et al., 2016).

Although CP-456,773 does not directly prevent kidney stone formation, it plays a crucial role in reducing renal inflammation, mitigating fibrosis, and preventing further tubular damage. By interrupting the inflammatory cycle driven by NLRP3 activation, CP-456,773 could indirectly slow nephrolithiasis progression, making it a valuable therapeutic candidate for inflammation-associated kidney stone disease.

#### 5.2.2 Targeting the NLRP3 inflammasome indirectly

Among pharmacological agents with anti-inflammatory properties, atorvastatin has shown therapeutic potential in calcium oxalate stone formers. While primarily known as a HMG-CoA reductase competitive inhibitor, atorvastatin also functions as an indirect inhibitor of the NLRP3 inflammasome, primarily by reducing oxidative stress and crystal deposition (Tsujihata, 2008; Joshi et al., 2015). Since CaOx crystals activate NLRP3 inflammasome via ROS/TXNIP pathway, atorvastatin helps mitigate oxidative stress, thereby reducing inflammation and preventing further stone aggregation (Tsujihata, 2008; Joshi et al., 2015). Beyond its role in lowering renal inflammation, atorvastatin is also used to reduce serum triglycerides and increase HDL levels, making it an effective treatment for hypercholesteremia, a condition that can secondarily contribute to nephrolithiasis. By addressing dyslipidemia, atorvastatin not only prevents further renal damage, but may also reduce the risk of recurrent kidney stone formation.

Another pharmacological agent of potential interest is phenylbutyric acid. Although it is an endoplasmic reticulum (ER) stress inhibitor and has not been directly implicated in the inhibition of NLRP3, phenylbutyric acid has been shown to reduce CaOx deposition and improve mitochondrial function in renal tubular epithelial cells. Additionally, it mitigates mitochondria-induced inflammation and ROS damage (Sharma et al., 2019). While its precise mechanism of action remains unclear, proposed models suggest that ER stress disrupts calcium homeostasis by altering calcium ion uptake and release. This imbalance leads to excessive calcium influx into the mitochondria, which in turn triggers ROS generation and disrupts the function of complex I/III and cytochrome c in the electron transport chain, ultimately impairing mitochondrial function. Mitochondrial dysfunction plays a key role in the activation of the NLRP3 inflammasome, a process that is further regulated by P2X7 receptor signaling (Figure 1).

Blocking P2X7 receptor activation, using ROS scavengers, is also an interesting pharmacological target. The P2X7 receptor is a ligand-gated ion channel activated by extracellular ATP, which is often released during cell stress, damage, or inflammation (Pelegrin, 2021). Its activation triggers NLRP3 inflammasome signaling through two primary pathways. First, activation of the P2X7 receptor leads to a sustained increase in intracellular calcium levels (Figure 1) by promoting calcium influx from the extracellular space and stimulating the release of calcium from the ER. This calcium overload damages mitochondria, causing the release of mtDNA, a known activator of the NLRP3 inflammasome (Gurcel et al., 2006). Second, P2X7 receptor activation reduces intracellular cAMP levels, which weakens cAMP-mediated inhibition of NLRP3 inflammasome activation. This process results in an increased abundance of the NLRP3 inflammasome, further driving inflammation and tissue damage (Rajamaki et al., 2013). Beyond its role in mitochondrial metabolism, mitochondria also regulate inflammatory cytokine expression through post-translational modifications and activation mechanisms. Persistent mitochondrial stress and ROS overproduction promote NLRP3 inflammasome assembly, leading to the secretion of pro-inflammatory cytokines IL-1β and IL-18. These cytokines exacerbate renal inflammation, ultimately contributing to tubular damage, fibrosis, and kidney stone formation (Figure 1). Given the interplay between mitochondrial dysfunction, ER stress, and P2X7 receptor signaling, targeting these pathways, through inhibitors like atorvastatin and phenylbutyric acid, may offer a therapeutic strategy to reduce inflammation and prevent kidney stone progression.

## 6 Discussion: conclusion and future directions

### 6.1 Summary of the role of NLRP3 in kidney stones and CKD

The NLRP3 inflammasome contributes to renal inflammation, kidney stone formation, and CKD progression. Its activation by calcium oxalate crystals, oxidative stress, and metabolic factors leads to IL-1β and IL-18 release, causing tubular injury, fibrosis, and stone recurrence. Chronic NLRP3 activity is linked to renal dysfunction and a higher risk of ESRD, making it a key therapeutic target.

### 6.2 Emerging therapies targeting NLRP3 for kidney stone prevention

New therapies target NLRP3 either directly, e.g., MCC950, CP-456,773, CY-09, or indirectly, through agents like atorvastatin and phenylbutyric acid that reduce oxidative stress and calcium oxalate deposition. Blocking P2X7 receptors, using ROS scavengers, and inhibiting caspase-1 also show promise. While preclinical findings are promising, clinical trials are necessary to establish safety and efficacy. Moreover, the long-term effects of NLRP3 inhibition remain uncertain, with potential risks including immunosuppression, impaired host defense, and unintended off-target immune responses (Vong et al., 2021; Tengesdal et al., 2024).

#### 6.2.1 Closing remarks

NLRP3 plays a critical role in both nephrolithiasis and CKD. Targeted inhibition may help reduce inflammation, prevent stone recurrence, and preserve kidney function. Continued research is essential to bring these therapies into clinical practice and improve patient outcomes. Future studies should evaluate the efficacy of direct NLRP3 inhibitors in kidney stone patients, as well as the long-term effects of indirect agents. Combination therapies, such as pairing NLRP3 inhibitors with antioxidants, or ER stress blockers, may offer synergistic benefits. Longitudinal research should also investigate whether targeting NLRP3 can slow CKD progression, particularly in high-risk populations.

## References

[B76] Anders H. J. Suarez-Alvarez B. Grigorescu M. Foresto-Neto O. Steiger S. Desai J. (2018). The macrophage phenotype and inflammasome component NLRP3 contributes to nephrocalcinosis-related chronic kidney disease independent from IL-1-mediated tissue injury. Kidney Int. 93 (3), 656–669. 10.1016/j.kint.2017.09.022 29241624

[B1] Asselman M. Verhulst A. Van Ballegooijen E. S. Bangma C. H. Verkoelen C. F. De Broe M. E. (2005). Hyaluronan is apically secreted and expressed by proliferating or regenerating renal tubular cells. Kidney Int. 68 (1), 71–83. 10.1111/j.1523-1755.2005.00382.x 15954897

[B2] Boxer M. B. Shen M. Auld D. S. Wells J. A. Thomas C. J. (2010). “A small molecule inhibitor of Caspase 1,” in Probe reports from the NIH molecular libraries Program (Bethesda, MD: National Center for Biotechnology Information). 21735610

[B3] Cao Y. Liu W. Hui L. Zhao J. Yang X. Wang Y. (2016). Renal tubular injury induced by ischemia promotes the formation of calcium oxalate crystals in rats with hyperoxaluria. Urolithiasis 44 (5), 389–397. 10.1007/s00240-016-0876-7 27040948

[B4] Chaiyarit S. Thongboonkerd V. (2020). Mitochondrial dysfunction and kidney stone disease. Front. Physiol. 11, 566506. 10.3389/fphys.2020.566506 33192563 PMC7606861

[B5] Chen Y. Yang S. Kong H. Wang Q. Chen S. Wang X. (2023). Oxalate-induced renal pyroptotic injury and crystal formation mediated by NLRP3-GSDMD signaling *in vitro* and *in vivo* . Mol. Med. Rep. 28 (5), 209. 10.3892/mmr.2023.13096 37732544 PMC10540023

[B6] Coe F. L. Parks J. H. Asplin J. R. (1992). The pathogenesis and treatment of kidney stones. N. Engl. J. Med. 327 (16), 1141–1152. 10.1056/NEJM199210153271607 1528210

[B7] Coll R. C. Robertson A. A. Chae J. J. Higgins S. C. Munoz-Planillo R. Inserra M. C. (2015). A small-molecule inhibitor of the NLRP3 inflammasome for the treatment of inflammatory diseases. Nat. Med. 21 (3), 248–255. 10.1038/nm.3806 25686105 PMC4392179

[B8] Crivelli J. J. Maalouf N. M. Paiste H. J. Wood K. D. Hughes A. E. Oates G. R. (2021). Disparities in kidney stone disease: a scoping review. J. Urol. 206 (3), 517–525. 10.1097/JU.0000000000001846 33904797 PMC8355087

[B9] Daudon M. Jungers P. Bazin D. Williams J. C. Jr. (2018). Recurrence rates of urinary calculi according to stone composition and morphology. Urolithiasis 46 (5), 459–470. 10.1007/s00240-018-1043-0 29392338 PMC6711148

[B10] Deguchi R. Komori T. Yamashita S. Hisaoka T. Kajimoto M. Kohjimoto Y. (2024). Suppression of renal crystal formation, inflammation, and fibrosis by blocking oncostatin M receptor beta signaling. Sci. Rep. 14 (1), 28913. 10.1038/s41598-024-80411-4 39572752 PMC11582566

[B11] Ding T. Zhao T. Li Y. Liu Z. Ding J. Ji B. (2021). Vitexin exerts protective effects against calcium oxalate crystal-induced kidney pyroptosis *in vivo* and *in vitro* . Phytomedicine 86, 153562. 10.1016/j.phymed.2021.153562 33857849

[B12] Du C. Wang X. Chen H. (2014). “Oxidative stress to renal tubular epithelial cells – a common pathway in renal pathologies,” in Systems biology of free radicals and antioxidants. Editor Laher I. (Berlin, Heidelberg: Springer Berlin Heidelberg), 2605–2624.

[B13] Fernandes-Alnemri T. Wu J. Yu J. W. Datta P. Miller B. Jankowski W. (2007). The pyroptosome: a supramolecular assembly of ASC dimers mediating inflammatory cell death via caspase-1 activation. Cell Death Differ. 14 (9), 1590–1604. 10.1038/sj.cdd.4402194 17599095 PMC3345951

[B14] Fong-Ngern K. Vinaiphat A. Thongboonkerd V. (2017). Microvillar injury in renal tubular epithelial cells induced by calcium oxalate crystal and the protective role of epigallocatechin-3-gallate. FASEB J. 31 (1), 120–131. 10.1096/fj.201600543R 27825102

[B15] Fu J. Wu H. (2023). Structural mechanisms of NLRP3 inflammasome assembly and activation. Annu. Rev. Immunol. 41, 301–316. 10.1146/annurev-immunol-081022-021207 36750315 PMC10159982

[B16] Fu R. Guo C. Wang S. Huang Y. Jin O. Hu H. (2017). Podocyte activation of NLRP3 inflammasomes contributes to the development of proteinuria in lupus nephritis. Arthritis Rheumatol. 69 (8), 1636–1646. 10.1002/art.40155 28544564 PMC5568813

[B17] Gambaro G. Croppi E. Bushinsky D. Jaeger P. Cupisti A. Ticinesi A. (2017). The risk of chronic kidney disease associated with urolithiasis and its urological treatments: a review. J. Urol. 198 (2), 268–273. 10.1016/j.juro.2016.12.135 28286070

[B18] Goldfarb S. (1988). Dietary factors in the pathogenesis and prophylaxis of calcium nephrolithiasis. Kidney Int. 34 (4), 544–555. 10.1038/ki.1988.216 3199675

[B19] Granata S. Masola V. Zoratti E. Scupoli M. T. Baruzzi A. Messa M. (2015). NLRP3 inflammasome activation in dialyzed chronic kidney disease patients. PLoS One 10 (3), e0122272. 10.1371/journal.pone.0122272 25798846 PMC4370586

[B20] Gritsenko A. Yu S. Martin-Sanchez F. Diaz-Del-Olmo I. Nichols E. M. Davis D. M. (2020). Priming is dispensable for NLRP3 inflammasome activation in human monocytes *in vitro* . Front. Immunol. 11, 565924. 10.3389/fimmu.2020.565924 33101286 PMC7555430

[B21] Gurcel L. Abrami L. Girardin S. Tschopp J. van der Goot F. G. (2006). Caspase-1 activation of lipid metabolic pathways in response to bacterial pore-forming toxins promotes cell survival. Cell 126 (6), 1135–1145. 10.1016/j.cell.2006.07.033 16990137

[B22] Hochheiser I. V. Pilsl M. Hagelueken G. Moecking J. Marleaux M. Brinkschulte R. (2022). Structure of the NLRP3 decamer bound to the cytokine release inhibitor CRID3. Nature 604 (7904), 184–189. 10.1038/s41586-022-04467-w 35114687

[B23] Hoffman A. Braun M. M. Khayat M. (2021). Kidney disease: kidney stones. FP Essent. 509, 33–38. 34643363

[B24] Huang G. Zhang Y. Zhang Y. Ma Y. (2023). Chronic kidney disease and NLRP3 inflammasome: pathogenesis, development and targeted therapeutic strategies. Biochem. Biophys. Rep. 33, 101417. 10.1016/j.bbrep.2022.101417 36620089 PMC9813680

[B25] Huang Y. Jiang H. Chen Y. Wang X. Yang Y. Tao J. (2018). Tranilast directly targets NLRP3 to treat inflammasome-driven diseases. EMBO Mol. Med. 10 (4), e8689. 10.15252/emmm.201708689 29531021 PMC5887903

[B26] Islamuddin M. Qin X. (2024). Renal macrophages and NLRP3 inflammasomes in kidney diseases and therapeutics. Cell Death Discov. 10 (1), 229. 10.1038/s41420-024-01996-3 38740765 PMC11091222

[B27] Jeong I. G. Kang T. Bang J. K. Park J. Kim W. Hwang S. S. (2011). Association between metabolic syndrome and the presence of kidney stones in a screened population. Am. J. Kidney Dis. 58 (3), 383–388. 10.1053/j.ajkd.2011.03.021 21620546

[B28] Jia Q. Huang Z. Wang G. Sun X. Wu Y. Yang B. (2022). Osteopontin: an important protein in the formation of kidney stones. Front. Pharmacol. 13, 1036423. 10.3389/fphar.2022.1036423 36452224 PMC9703462

[B29] Joshi S. Wang W. Peck A. B. Khan S. R. (2015). Activation of the NLRP3 inflammasome in association with calcium oxalate crystal induced reactive oxygen species in kidneys. J. Urol. 193 (5), 1684–1691. 10.1016/j.juro.2014.11.093 25437532 PMC4406847

[B30] Jungers P. Joly D. Barbey F. Choukroun G. Daudon M. (2004). ESRD caused by nephrolithiasis: prevalence, mechanisms, and prevention. Am. J. Kidney Dis. 44 (5), 799–805. 10.1016/s0272-6386(04)01131-x 15492945

[B31] Kim J. Y. Lee J. K. Park J. T. Chang T. I. (2024). Risk of incident chronic kidney disease among patients with urolithiasis: a nationwide longitudinal cohort study. Clin. Kidney J. 17 (3), sfae030. 10.1093/ckj/sfae030 38435351 PMC10906355

[B32] Kitching A. R. Turner A. L. Wilson G. R. Semple T. Odobasic D. Timoshanko J. R. (2005). IL-12p40 and IL-18 in crescentic glomerulonephritis: IL-12p40 is the key Th1-defining cytokine chain, whereas IL-18 promotes local inflammation and leukocyte recruitment. J. Am. Soc. Nephrol. 16 (7), 2023–2033. 10.1681/ASN.2004121075 15888563

[B33] Lamkanfi M. Mueller J. L. Vitari A. C. Misaghi S. Fedorova A. Deshayes K. (2009). Glyburide inhibits the Cryopyrin/Nalp3 inflammasome. J. Cell Biol. 187 (1), 61–70. 10.1083/jcb.200903124 19805629 PMC2762099

[B34] Lankadeva Y. R. May C. N. Bellomo R. Evans R. G. (2022). Role of perioperative hypotension in postoperative acute kidney injury: a narrative review. Br. J. Anaesth. 128 (6), 931–948. 10.1016/j.bja.2022.03.002 35465952

[B35] Lee J. K. Kim S. H. Lewis E. C. Azam T. Reznikov L. L. Dinarello C. A. (2004). Differences in signaling pathways by IL-1beta and IL-18. Proc. Natl. Acad. Sci. U. S. A. 101 (23), 8815–8820. 10.1073/pnas.0402800101 15161979 PMC423278

[B36] Leslie S. W. Sajjad H. Murphy P. B. (2025). “Renal calculi, nephrolithiasis,” in StatPearls Florida, United States: StatPearls Publishing. 28723043

[B37] Liu J. Huang J. Gong B. Cheng S. Liu Y. Chen Y. (2023). Polydatin protects against calcium oxalate crystal-induced renal injury through the cytoplasmic/mitochondrial reactive oxygen species-NLRP3 inflammasome pathway. Biomed. Pharmacother. 167, 115621. 10.1016/j.biopha.2023.115621 37793278

[B38] Liu T. Zhang L. Joo D. Sun S. C. (2017). NF-κB signaling in inflammation. Signal Transduct. Target Ther. 2, 17023. 10.1038/sigtrans.2017.23 29158945 PMC5661633

[B39] Ludwig-Portugall I. Bartok E. Dhana E. Evers B. D. Primiano M. J. Hall J. P. (2016). An NLRP3-specific inflammasome inhibitor attenuates crystal-induced kidney fibrosis in mice. Kidney Int. 90 (3), 525–539. 10.1016/j.kint.2016.03.035 27262364

[B40] Malik A. Kanneganti T. D. (2018). Function and regulation of IL-1α in inflammatory diseases and cancer. Immunol. Rev. 281 (1), 124–137. 10.1111/imr.12615 29247991 PMC5739076

[B41] McKee C. M. Coll R. C. (2020). NLRP3 inflammasome priming: a riddle wrapped in a mystery inside an enigma. J. Leukoc. Biol. 108 (3), 937–952. 10.1002/JLB.3MR0720-513R 32745339

[B42] Minisola S. Gianotti L. Bhadada S. Silverberg S. J. (2018). Classical complications of primary hyperparathyroidism. Best. Pract. Res. Clin. Endocrinol. Metab. 32 (6), 791–803. 10.1016/j.beem.2018.09.001 30665547

[B43] Mulay S. R. Kulkarni O. P. Rupanagudi K. V. Migliorini A. Darisipudi M. N. Vilaysane A. (2013). Calcium oxalate crystals induce renal inflammation by NLRP3-mediated IL-1β secretion. J. Clin. Invest. 123 (1), 236–246. 10.1172/JCI63679 23221343 PMC3533282

[B44] Munoz-Planillo R. Kuffa P. Martinez-Colon G. Smith B. L. Rajendiran T. M. Nunez G. (2013). K⁺ efflux is the common trigger of NLRP3 inflammasome activation by bacterial toxins and particulate matter. Immunity 38 (6), 1142–1153. 10.1016/j.immuni.2013.05.016 23809161 PMC3730833

[B45] Muschialli L. Mannath A. Moochhala S. H. Shroff R. Ferraro P. M. (2024). Epidemiological and biological associations between cardiovascular disease and kidney stone formation: a systematic review and meta-analysis. Nutr. Metab. Cardiovasc Dis. 34 (3), 559–568. 10.1016/j.numecd.2023.09.011 38431384

[B46] Oliveira C. B. Lima C. A. D. Vajgel G. Sandrin-Garcia P. (2021). The role of NLRP3 inflammasome in lupus nephritis. Int. J. Mol. Sci. 22 (22), 12476. 10.3390/ijms222212476 34830358 PMC8625721

[B47] Pak C. Y. Sakhaee K. Moe O. W. Poindexter J. Adams-Huet B. Pearle M. S. (2011). Defining hypercalciuria in nephrolithiasis. Kidney Int. 80 (7), 777–782. 10.1038/ki.2011.227 21775970 PMC4354881

[B48] Pearle M. S. Calhoun E. A. Curhan G. C. Urologic Diseases of America P. (2005). Urologic diseases in America project: urolithiasis. J. Urol. 173 (3), 848–857. 10.1097/01.ju.0000152082.14384.d7 15711292

[B49] Pelegrin P. (2021). P2X7 receptor and the NLRP3 inflammasome: partners in crime. Biochem. Pharmacol. 187, 114385. 10.1016/j.bcp.2020.114385 33359010

[B50] Perregaux D. G. McNiff P. Laliberte R. Hawryluk N. Peurano H. Stam E. (2001). Identification and characterization of a novel class of interleukin-1 post-translational processing inhibitors. J. Pharmacol. Exp. Ther. 299 (1), 187–197. 10.1016/s0022-3565(24)29317-4 11561079

[B51] Primiano M. J. Lefker B. A. Bowman M. R. Bree A. G. Hubeau C. Bonin P. D. (2016). Efficacy and Pharmacology of the NLRP3 inflammasome inhibitor CP-456,773 (CRID3) in murine models of dermal and pulmonary inflammation. J. Immunol. 197 (6), 2421–2433. 10.4049/jimmunol.1600035 27521339

[B52] Rajamaki K. Nordstrom T. Nurmi K. Akerman K. E. Kovanen P. T. Oorni K. (2013). Extracellular acidosis is a novel danger signal alerting innate immunity via the NLRP3 inflammasome. J. Biol. Chem. 288 (19), 13410–13419. 10.1074/jbc.M112.426254 23530046 PMC3650379

[B53] Rudolphi K. Gerwin N. Verzijl N. van der Kraan P. van den Berg W. (2003). Pralnacasan, an inhibitor of interleukin-1beta converting enzyme, reduces joint damage in two murine models of osteoarthritis. Osteoarthr. Cartil. 11 (10), 738–746. 10.1016/s1063-4584(03)00153-5 13129693

[B54] Rule A. D. Bergstralh E. J. Melton L. J. 3rd Li X. Weaver A. L. Lieske J. C. (2009). Kidney stones and the risk for chronic kidney disease. Clin. J. Am. Soc. Nephrol. 4 (4), 804–811. 10.2215/CJN.05811108 19339425 PMC2666438

[B75] Rumora L. Hlapcic I. Hulina-Tomaskovic A. Somborac-Bacura A. Bosnar M. Rajkovic M. G. (2021). Pathogen-associated molecular patterns and extracellular Hsp70 interplay in NLRP3 inflammasome activation in monocytic and bronchial epithelial cellular models of COPD exacerbations. APMIS 129 (2), 80–90. 10.1111/apm.13089 33022793

[B55] Schroder K. Tschopp J. (2010). The inflammasomes. Cell 140 (6), 821–832. 10.1016/j.cell.2010.01.040 20303873

[B56] Sharma M. Naura A. S. Singla S. K. (2019). Modulatory effect of 4-phenyl butyric acid on hyperoxaluria-induced renal injury and inflammation. Mol. Cell Biochem. 451 (1-2), 185–196. 10.1007/s11010-018-3405-x 30066041

[B57] Shastri S. Patel J. Sambandam K. K. Lederer E. D. (2023). Kidney stone pathophysiology, evaluation and management: core curriculum 2023. Am. J. Kidney Dis. 82 (5), 617–634. 10.1053/j.ajkd.2023.03.017 37565942 PMC11370273

[B58] Smarick S. (2009). “Urine output,” in Small animal critical care medicine. Editors Silverstein D. C. Hopper K. (W.B. Saunders), 865–868.

[B59] Stamatelou K. Goldfarb D. S. (2023). Epidemiology of kidney stones. Healthc. (Basel) 11 (3), 424. 10.3390/healthcare11030424 PMC9914194 36766999

[B60] Sun X. Y. Gan Q. Z. Ouyang J. M. (2015). Calcium oxalate toxicity in renal epithelial cells: the mediation of crystal size on cell death mode. Cell Death Discov. 1, 15055. 10.1038/cddiscovery.2015.55 27551481 PMC4979418

[B61] Sun X. Y. Xu M. Ouyang J. M. (2017). Effect of crystal shape and aggregation of calcium oxalate monohydrate on cellular toxicity in renal epithelial cells. ACS Omega 2 (9), 6039–6052. 10.1021/acsomega.7b00510 30023760 PMC6044778

[B62] Sun Y. Liu Y. Guan X. Kang J. Wang X. Liu Q. (2020). Atorvastatin inhibits renal inflammatory response induced by calcium oxalate crystals via inhibiting the activation of TLR4/NF-κB and NLRP3 inflammasome. IUBMB Life 72 (5), 1065–1074. 10.1002/iub.2250 32083808

[B63] Sun Y. Sun H. Zhang Z. Tan F. Qu Y. Lei X. (2024). New insight into oxidative stress and inflammatory responses to kidney stones: potential therapeutic strategies with natural active ingredients. Biomed. Pharmacother. 179, 117333. 10.1016/j.biopha.2024.117333 39243436

[B64] Swanson K. V. Deng M. Ting J. P. (2019). The NLRP3 inflammasome: molecular activation and regulation to therapeutics. Nat. Rev. Immunol. 19 (8), 477–489. 10.1038/s41577-019-0165-0 31036962 PMC7807242

[B65] Tengesdal I. W. Banks M. Dinarello C. A. Marchetti C. (2024). Screening NLRP3 drug candidates in clinical development: lessons from existing and emerging technologies. Front. Immunol. 15, 1422249. 10.3389/fimmu.2024.1422249 39188718 PMC11345644

[B66] Tsujihata M. (2008). Mechanism of calcium oxalate renal stone formation and renal tubular cell injury. Int. J. Urol. 15 (2), 115–120. 10.1111/j.1442-2042.2007.01953.x 18269444

[B67] Vong C. T. Tseng H. H. L. Yao P. Yu H. Wang S. Zhong Z. (2021). Specific NLRP3 inflammasome inhibitors: promising therapeutic agents for inflammatory diseases. Drug Discov. Today 26 (6), 1394–1408. 10.1016/j.drudis.2021.02.018 33636340

[B68] Wang K. Ge J. Han W. Wang D. Zhao Y. Shen Y. (2022). Risk factors for kidney stone disease recurrence: a comprehensive meta-analysis. BMC Urol. 22 (1), 62. 10.1186/s12894-022-01017-4 35439979 PMC9017041

[B69] Wigner P. Grebowski R. Bijak M. Szemraj J. Saluk-Bijak J. (2021). The molecular aspect of nephrolithiasis development. Cells 10 (8), 1926. 10.3390/cells10081926 34440695 PMC8393760

[B70] Worcester E. M. Coe F. L. (2008). New insights into the pathogenesis of idiopathic hypercalciuria. Semin. Nephrol. 28 (2), 120–132. 10.1016/j.semnephrol.2008.01.005 18359393 PMC2362396

[B71] Xu Y. Li G. Liu W. Ge D. Hao Z. Wang W. (2025). Inhibition of NLRP3 alleviates calcium oxalate crystal-induced renal fibrosis and crystal adhesion. Urolithiasis 53 (1), 44. 10.1007/s00240-025-01716-1 40035889

[B72] Zahid A. Li B. Kombe A. J. K. Jin T. Tao J. (2019). Pharmacological inhibitors of the NLRP3 inflammasome. Front. Immunol. 10, 2538. 10.3389/fimmu.2019.02538 31749805 PMC6842943

[B73] Zheng Z. Xu K. Li C. Qi C. Fang Y. Zhu N. (2021). NLRP3 associated with chronic kidney disease progression after ischemia/reperfusion-induced acute kidney injury. Cell Death Discov. 7 (1), 324. 10.1038/s41420-021-00719-2 34716316 PMC8556399

[B74] Zisman A. L. Evan A. P. Coe F. L. Worcester E. M. (2015). Do kidney stone formers have a kidney disease? Kidney Int. 88 (6), 1240–1249. 10.1038/ki.2015.254 26376133 PMC4675687

